# Niche Segregation Between Black‐Necked Crane and Common Crane in Human‐Modified Landscape, Huize Black‐Necked Crane National Nature Reserve, Yunnan Province, Southwestern China

**DOI:** 10.1002/ece3.73402

**Published:** 2026-04-20

**Authors:** Jiayi Wu, Yuhui Si, Xiaoxuan Shang, Chao Li, Qiang Liu

**Affiliations:** ^1^ College of Forestry Southwest Forestry University Kunming Yunnan China; ^2^ College of Ecology and Environment (College of Wetlands) Southwest Forestry University Kunming Yunnan China; ^3^ Yunnan Key Laboratory of Plateau Wetland Conservation Restoration and Ecological Services Kunming China

**Keywords:** Black‐necked Crane, Common Crane, diet analysis, dietary diversity, fecal DNA metabarcoding, high‐throughput sequencing

## Abstract

Agricultural expansion has created mosaic landscapes that are crucial for migratory birds, yet these anthropogenic habitats intensify challenges like interspecific competition. Understanding how sympatric species coexist through nutritional niche adjustments is a critical knowledge gap for conservation. We studied the wintering Black‐necked Crane ( *Grus nigricollis* ) and Common Crane ( *G. grus* ) in an agro‐wetland system in southwest China, where seasonal resource decline is expected to drive niche differentiation. Using fecal DNA metabarcoding, we reconstructed and compared their plant‐based diets throughout the winter. The two species employed divergent foraging strategies. Black‐necked Cranes were conservative generalists, maintaining high dietary richness and consistently relying on a core set of natural food sources (e.g., Poaceae, Solanaceae, and Cyperaceae), using crops only as supplements. Conversely, Common Cranes were dynamic specialists, exhibiting a complete turnover of preferred foods and opportunistically concentrating on the most energy‐dense crops available in each period (e.g., *Fagopyrum esculentum* , *Helianthus annuus* ). This strategic divergence resulted in a continuous decrease in NO, from high initial similarity to complete partitioning by the pre‐migration stage, while ND increased correspondingly, reflecting progressive trophic niche separation. We conclude that this dynamic niche partitioning, driven by distinct responses to seasonal resource availability, is the primary mechanism facilitating the coexistence of these two large congeneric species. Our findings reveal profound dietary plasticity and offer a scientific basis for managing anthropogenic landscapes to support multispecies conservation goals.

## Graphical abstract

We investigated how Black‐necked Cranes and Common Cranes coexist in agro‐wetland landscapes of southwest China by reconstructing their plant‐based diets using eDNA metabarcoding. Black‐necked Cranes maintained broad, stable diets centered on natural vegetation, while Common Cranes shifted dynamically to energy‐dense crops, leading to progressive niche divergence. This dietary plasticity highlights dynamic niche partitioning as a key mechanism of coexistence and provides insights for conservation in human‐modified habitats.

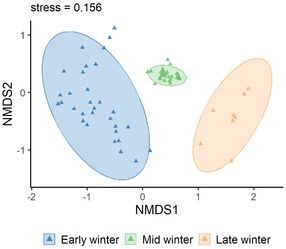

## 1 Introduction

The accelerating loss of biodiversity is a defining global challenge, driven primarily by anthropogenic landscape modification through agricultural expansion and intensification (Kehoe et al. 2017; Osumanu and Kosoe 2023). Paradoxically, these same agricultural mosaics are becoming de facto refuges for many wildlife species, particularly migratory birds that depend on them during the nonbreeding season (Langendoen et al. 2021). However, such anthropogenic systems introduce significant ecological pressures, including intensified interspecific competition and resource uncertainty (Andersson et al. 2021; Marchesan and Kolasa 2024). This is especially critical along the East Asian–Australasian Flyway, where agricultural landscapes sustain vast waterbird populations. Among these, cranes (Gruidae) are one of the world's most threatened avian families, making the study of their adaptation to these novel ecosystems a conservation priority (Li et al. 2019; BirdLife International 2023). The Black‐necked Crane ( *Grus nigricollis* ) is classified as Near Threatened and is a flagship species, breeding on the Qinghai–Tibetan Plateau and wintering mainly in Yunnan, Guizhou, and Tibet (IUCN 2023; Jiang et al. 2025). The Common Crane ( *G. grus* ), listed as Least Concern, has a broad Palearctic distribution but has recently expanded its wintering range into southwest China, now occurring in major Black‐necked Crane wintering regions such as Napahai, Lashihai, and Caohai (Kong et al. 2018; Zhang et al. 2021; IUCN 2023; Yan et al. 2025).

This study focuses on the Black‐necked Crane, a flagship species for high‐altitude wetland conservation. With a global population of around 17,000, its wintering populations in southwest China have increasingly shifted from natural wetlands to agricultural fields, heightening their dependence on human‐dominated landscapes (Kong et al. 2011; Wu et al. 2020). In contrast, the Common Crane has shown a marked and rapid increase in wintering numbers across southwest China over the past two decades, with several sites now supporting populations that match or exceed those of the Black‐necked Crane (Kong et al. 2018; Zhang et al. 2021; Li et al. 2022; Wang, Zhan, et al. 2024). Compounding this precarity is the recent and rapid population expansion of the ecologically flexible Common Crane at key Black‐necked Crane wintering sites. This sympatric expansion has intensified interspecific competition, as both species now heavily overlap in their use of agricultural residues like *Solanum tuberosum* , *Zea mays* , and *Fagopyrum esculentum* (Kong et al. 2018; Jia et al. 2019). At Huize National Nature Reserve, the wintering Black‐necked Crane population has remained relatively stable (c. 152–187 individuals, 2007–2024), while the Common Crane population has more than doubled (c. 150–311 individuals) over the same period, further heightening the likelihood of competition as both species increasingly converge on the same croplands and wetland margins (Qiu and Yang 2012; Kong et al. 2018; Liu 2024).

Although previous studies have recognized spatial partitioning as a mechanism for species coexistence, the role and dynamic changes of dietary nutritional niche differentiation under food resource scarcity and interspecific competition have not been systematically investigated, leaving a critical knowledge gap (Dong et al. 2016, 2025; Hou, Pan, et al. 2021). Here, we investigate the variation in diet of coexisting Black‐necked and Common Cranes at the Huize National Nature Reserve in Yunnan, China—a vital agro‐wetland mosaic where the competitive interface between these species is pronounced. To elucidate the mechanisms of their coexistence, we employed high‐resolution fecal DNA metabarcoding. This noninvasive approach overcomes the taxonomic and quantitative limitations of traditional methods, allowing for precise, stage specific tracking of dietary composition and niche variation throughout the wintering period (ter Schure et al. 2020; Mas‐Carrió et al. 2024).

We hypothesized that Black‐necked Cranes employ nutritional niche differentiation to mitigate competition from Common Cranes, particularly in the winter stage when food becomes limited. This central hypothesis leads to three specific objectives for this study: (1) to characterize the stage specific dietary composition of both crane species using fecal DNA metabarcoding; (2) to quantify temporal variation in their nutritional niche width and overlap to reveal patterns of dietary partitioning; and (3) to elucidate the adaptive foraging strategies that facilitate their coexistence. By addressing these objectives, our study provides direct, high‐resolution evidence for niche‐based coexistence theory and offers critical scientific guidance for managing food resources and habitats in anthropogenic landscapes to support sympatric species.

## 2 Methods

### 2.1 Study Area

This study was conducted in the Nianhu area (E103°12′–103°22′, N26°38′–26°44′) within the Huize Black‐necked Crane National Nature Reserve, Yunnan Province, southwestern China. Situated on the Yunnan‐Guizhou Plateau, the reserve is a crucial wintering and stopover site for migratory waterbirds along the East Asian–Australasian Flyway. The study area covers approximately 9076.28 ha, including a water surface of 1330.00 ha, and experiences a subtropical monsoon climate with a mean annual temperature of 9.6°C. Its landscape is a habitat mosaic, comprising natural wetlands, meadows, and forests interspersed with man‐made farmlands. Local agriculture is dominated by the dryland cultivation of potatoes, buckwheat, and oilseed crops. The post‐harvest residues from these fields provide a critical anthropogenic food subsidy for wintering cranes (Qiu and Yang 2012). Consequently, Nianhu serves as a key sympatric wintering ground for the Black‐necked Crane and the Common Crane. By 2024, Black‐necked Crane and Common Crane populations had reached 187 and 311 individuals, respectively (Liu 2024). The shared reliance of both species on this finite agricultural resource offers an ideal natural experiment to investigate their foraging ecology, interspecific competition, and coexistence mechanisms.

### 2.2 Sample Collection

**FIGURE 1:**
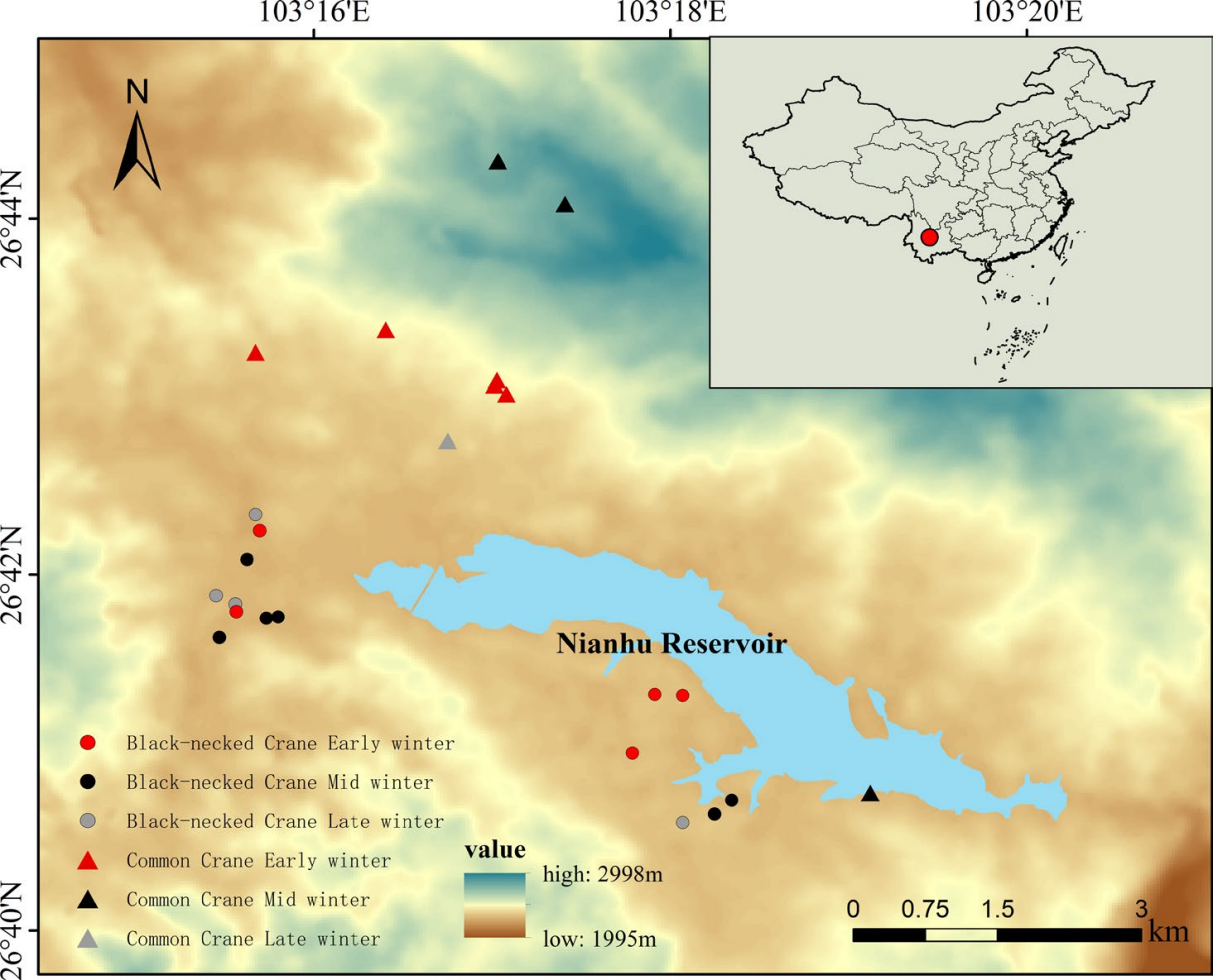
Locations of the 15 Black‐necked Crane sampling sites and eight Common Crane sampling sites in the Nianhu area of the Huize National Nature Reserve. Black‐necked Cranes are represented by circles in red (early winter), black (mid‐winter), and gray (late winter), whereas Common Cranes are shown as triangles in red (early winter), black (mid‐winter), and gray (late winter).

Fresh fecal samples of Black‐necked Cranes and Common Cranes were collected from 29 November 2024 to 14 March 2025. Because fecal freshness strongly affects DNA quality and downstream extraction, PCR, and sequencing (Oehm et al. 2017), we followed a standardized sampling protocol. We first located small, species‐specific foraging groups in the field. After observing the birds for 15–30 min and waiting for them to leave naturally, we approached the foraging site and searched for fresh feces that showed visible mucous layers and moisture, together with clear pecking traces to ensure species identity. Each sample was placed in a sterile cryovial and temporarily preserved in a portable ice box before being transferred to the laboratory the same day and stored at −80°C until processing. To represent different phases of the wintering period—early (November), mid (January), and late (March)—we collected 97 fecal samples from Black‐necked Cranes (early: 32; mid: 31; late: 34) and 68 from Common Cranes (early: 30; mid: 30; late: 8) (Figure 1).

**FIGURE 2:**
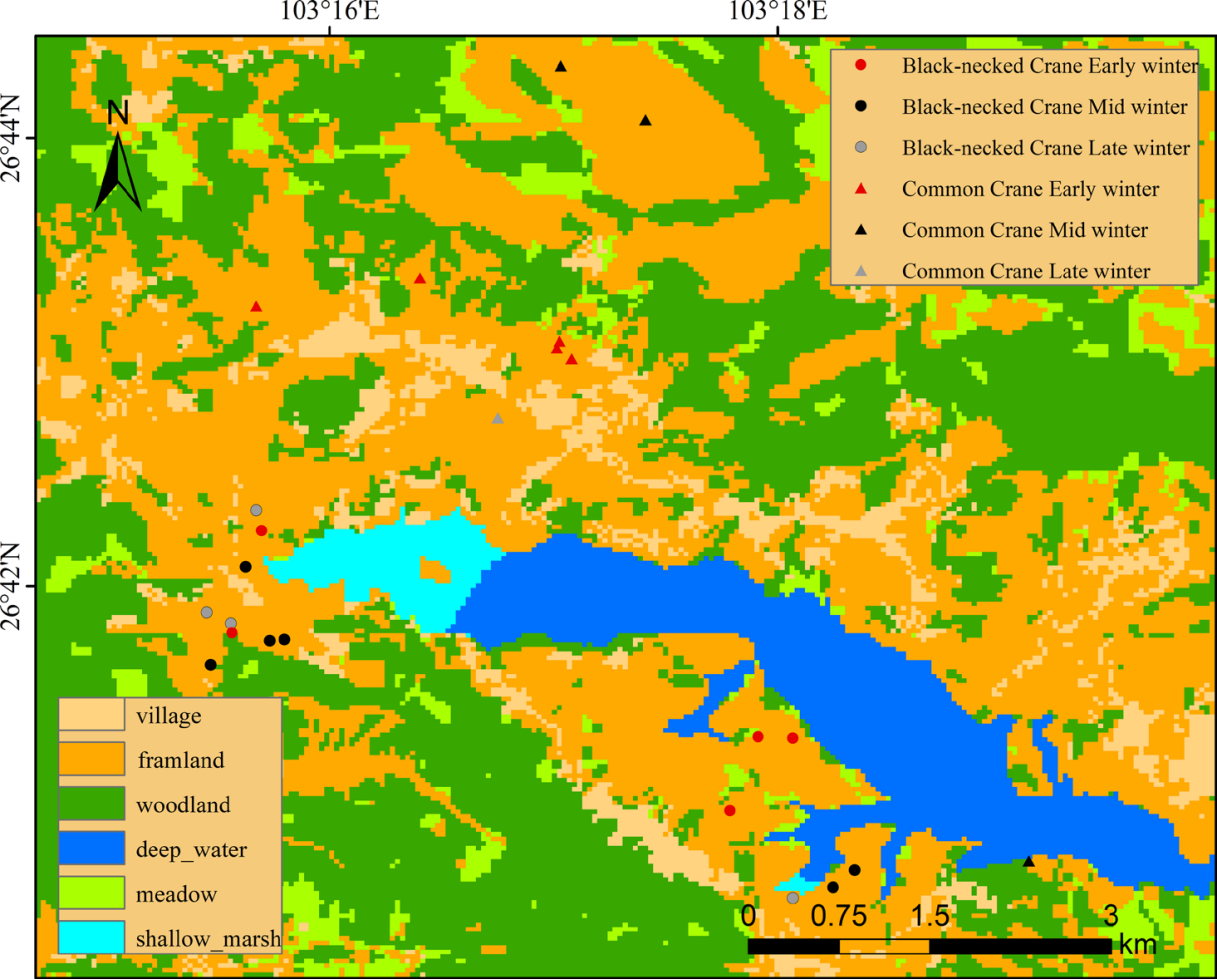
Land‐cover types and seasonal locations of Black‐necked Cranes and Common Cranes in the Nianhu area of the Huize National Nature Reserve. Land‐cover categories include village, farmland, woodland, deep water, meadow, and shallow marsh. Black‐necked Crane sites are shown as circles in red (early winter), black (mid‐winter), and gray (late winter), whereas Common Crane sites are represented by triangles in red (early winter), black (mid‐winter), and gray (late winter).

Black‐necked Crane samples were primarily collected from farmland areas surrounding Nianhu (Figure 2). Early‐winter samples were obtained from five sites at 2500.6 ± 9.45 m. Mid‐winter samples came from six sites at 2496 ± 4.47 m, and late‐winter samples from four sites at 2492.75 ± 2.36 m. Sampling elevations for Common Cranes were considerably higher. In early winter, samples were collected from four mid‐slope sites on three mountains surrounding Nianhu, at 2597.2 ± 35.7 m. Mid‐winter samples were obtained from three sites (mean 2735 ± 213 m), including one farmland site near Nianhu (2490 m) and two high‐altitude sites near mountaintops (mean 2827 m) (Figure 2). Late‐winter samples were collected from farmland located in a valley between two mountains at 2528 m (Figure 2). All samples were collected along pre‐established transects using dispersed search methods to ensure spatial representativeness.

### 2.3 DNA Extraction, Sequencing and Bioinformatics

To characterize the plant‐based diet of the study species, we used the chloroplast gene rbcL as a molecular marker, owing to its broad universality and high discriminatory power for plants (Angiosperm Phylogeny Group 2009). Fecal DNA served as the template, and first‐round PCR amplification employed the universal chloroplast rbcL primers ZlaF (5′‐ATGTCACCACCACCAACAGAGACTAAAGC‐3′) and hp2R (5′‐CGTCCTTTGTAACGATCAAG‐3′) (Hofreiter et al. 2000). PCR reactions were performed in a 30 μL system containing 15 μL of 2× Hieff Robust PCR Master Mix, 1 μL each of forward and reverse primers (0.3 μmol/L), 10–20 ng of fecal DNA, and nuclease‐free water to a final volume of 9–12 μL. The thermocycling protocol included: initial denaturation at 94°C for 3 min; 5 cycles of 94°C for 30 s, 45°C for 20 s, and 65°C for 30 s; 20 cycles of 94°C for 20 s, 55°C for 20 s, and 72°C for 30 s; and a final extension at 72°C for 5 min. A second‐round PCR was conducted to incorporate Illumina bridge PCR–compatible adapters. The reaction volume was also 30 μL, consisting of 15 μL of 2× Hieff Robust PCR Master Mix, 1 μL each of forward and reverse primers (0.3 μmol/L), 20–30 ng of first‐round PCR product, and nuclease‐free water to 9–12 μL. Cycling conditions were: 95°C for 3 min; 5 cycles of 94°C for 20 s, 55°C for 20 s, and 72°C for 30 s; and a final extension at 72°C for 5 min. Library size was assessed using 2% agarose gel electrophoresis, and concentration was quantified with a Qubit 3.0 fluorometer. All samples were pooled in equimolar amounts (1:1). Amplicon library preparation and high‐throughput sequencing were conducted on the Illumina MiSeq platform (PE150) at Sangon Biotech (Shanghai) Co. Ltd.

Raw sequences generated by the sequencing company were processed with cutadapt to remove primer and adapter sequences, followed by paired‐end merging using PEAR. Quality filtering and dereplication were conducted with PRINSEQ to obtain high‐quality reads. Subsequently, OTU clustering was performed with Usearch: chimeric sequences were identified and removed, and the remaining high‐quality sequences were clustered at a 97% similarity threshold to generate OTU representative sequences (Edgar and Flyvbjerg 2015). Taxonomic assignments were obtained by querying OTU representative sequences against the GenBank database (NCBI) using BLAST, combined with classification through both the RDP classifier and SINTAX algorithms. Only matches with ≥ 90% sequence coverage and ≥ 90% similarity were considered reliable and retained for downstream taxonomic identification. To ensure accurate species‐level assignment, taxonomic results were cross‐referenced with species distribution records from *the Huize Black‐necked Crane National Nature Reserve* flora checklist (Qiu and Yang 2012). Based on these resources, plant taxa detected in the fecal metabarcoding dataset were filtered to confirm their occurrence within the study area. Owing to the limited resolution of the rbcL marker within certain genera, some dietary items could only be assigned at the genus level.

Metabarcoding of 97 Black‐necked Crane fecal samples produced 2081 OTU representative sequences. After stringent quality filtering and taxonomic validation, 979 effective OTUs were retained, representing 71 dietary categories, 65 identified to species level. For Common Cranes, sequencing of 68 fecal samples produced 1413 OTU representative sequences. Following the same filtering and validation workflow, 672 OTUs were retained, representing 63 dietary categories, including 56 identified to species level. The species accumulation curves reached clear asymptotes in the early (*n* = 20), mid (*n* = 6), and late (*n* = 6) winter periods, indicating that sampling depth was sufficient within each period.

### 2.4 Statistical Analyses

To evaluate sampling effort and sequencing depth, we generated species accumulation curves using the function specaccum of the R package vegan (Oksanen et al. 2012). Dietary preferences were quantified using two standardized indices: (1) Relative Read Abundance (RRA), defined as the proportion of reads assigned to a given dietary OTU relative to the total dietary reads within each fecal sample, and (2) Food OTU Richness, defined as the number of distinct dietary OTUs detected per sample (Kilburn et al. 2020). Within‐group dietary diversity was assessed by calculating α‐diversity indices, including species richness, Shannon diversity, Simpson diversity, and Pielou's evenness (Spellerberg and Fedor 2003), implemented with the R packages vegan and permute (Oksanen et al. 2012). Nutritional niche metrics were quantified using NO (Schoener's niche overlap index), ND (1 − NO) and niche breadth to evaluate overall resource utilization and the degree of trophic niche divergence between the two crane species. β‐diversity was analyzed using Bray–Curtis dissimilarity matrices, and dietary composition differences were visualized through nonmetric multidimensional scaling (NMDS) with the function metaMDS of the R package vegan (Minchin 1987). Elevation of sampling sites and dietary composition metrics were summarized as mean ± standard deviation (SD).

### 2.5 Land‐Cover Classification

We downloaded a Landsat 8 satellite image (acquired in November 2024) from the USGS Earth Explorer platform (https://earthexplorer.usgs.gov/) and used it as the primary data source. The study area was clipped in ENVI 5.6.2, followed by standard preprocessing and supervised classification. Due to the spatial resolution limitations of the Landsat 8 imagery, high‐resolution Google Earth Pro images were used as auxiliary references for selecting training samples during the supervised classification. After classification, the resulting land‐cover map was imported into ArcMap, and the “Eliminate” tool was applied to remove small, isolated patches generated during the interpretation process. The classification achieved an overall accuracy of 97.79%, with a mean Kappa coefficient of 97.02%. Based on field surveys, the habitat types were categorized into seven classes: woodland, grassland, farmland, shallow marsh, deep water, and village. Specifically: (1) woodland—vegetation‐covered areas on mountains and slopes; (2) grassland—areas dominated by herbaceous vegetation; (3) farmland—areas cultivated with crops; (4) shallow marsh—wetland areas with water depth less than 50 cm; (5) deep water–water bodies with depth greater than 50 cm; and (6) village—areas containing buildings.

## 3 Results

### 3.1 Foraging Habitat Use of BNC and CC

The two crane species exhibited distinct elevational and spatial foraging patterns across the wintering period (Figures 1 and 2). Black‐necked Cranes consistently foraged within a narrow elevational band centered around 2490–2500 m, showing minimal vertical fluctuation throughout early, mid, and late winter. This stability was evident in both the point‐based elevation data (e.g., Black‐necked Cranes foraging sites clustered tightly around 2490–2516 m in winter) and the spatial distribution maps, where Black‐necked Cranes' locations remained concentrated in farmland patches adjacent to the Nianhu Reservoir margins.

In contrast, Common Cranes displayed a markedly broader elevational range (ca. 2490–2874 m), with clear seasonal shifts in vertical habitat use. During early winter, Common Cranes primarily occupied mid elevation farmland and meadow mosaics (approximately 2550–2650 m). Their elevational range expanded substantially in mid winter, which not only enabled them to reach the highest recorded foraging sites (up to 2840 and 2874 m), but also caused partial spatial overlap with Black‐necked Cranes at lower elevations while simultaneously extending into higher and more topographically variable terrain. By late winter, Common Cranes again concentrated around mid elevation zones (ca. 2550 m).

Spatially, the two species exhibited partial overlap but clear differentiation in core foraging areas. The distribution maps show Black‐necked Cranes concentrated in low‐lying farmland and shallow marsh mosaics near the reservoir, whereas Common Cranes were dispersed across a wider landscape, including higher elevation slopes and more distant agricultural valleys.

Together, these results indicate that Black‐necked Cranes maintain a stable, low elevation foraging strategy tightly linked to farmland adjacent to wetland roosts, whereas Common Cranes employ a more flexible, wide‐ranging strategy that varies seasonally and encompasses a substantially broader elevational gradient.

### 3.2 Dietary Spretrum and Variation in Diet of BNC and CC at Various Stages of Winter

**FIGURE 3:**
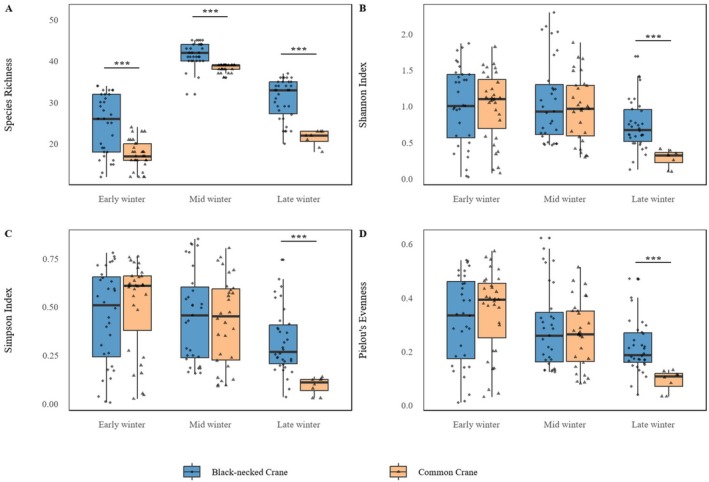
Changes in four α‐diversity metrics for Black‐necked Cranes and Common Cranes during early, mid, and late winter. Metrics include species richness (A), Shannon index (B), Simpson index (C), and Pielou's evenness (D). Asterisks (*) indicate significant differences between the two species.

**FIGURE 4:**
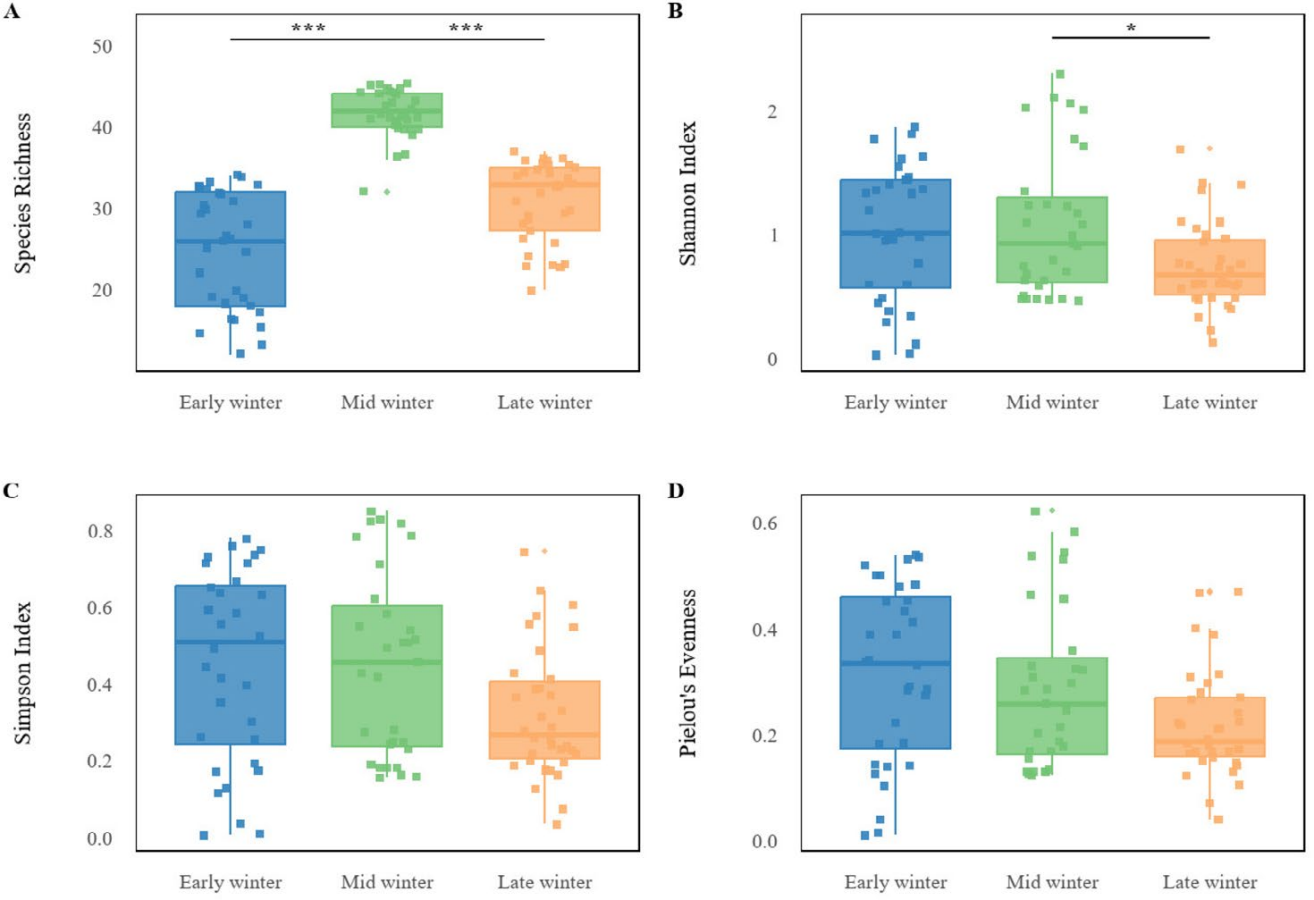
Temporal changes in four alpha‐diversity metrics for Black‐necked Cranes across the early, mid and late winter periods: (A) species richness; (B) Shannon index; (C) Simpson index; and (D) Pielou's evenness. Asterisks indicate statistically significant differences between groups.

**FIGURE 5:**
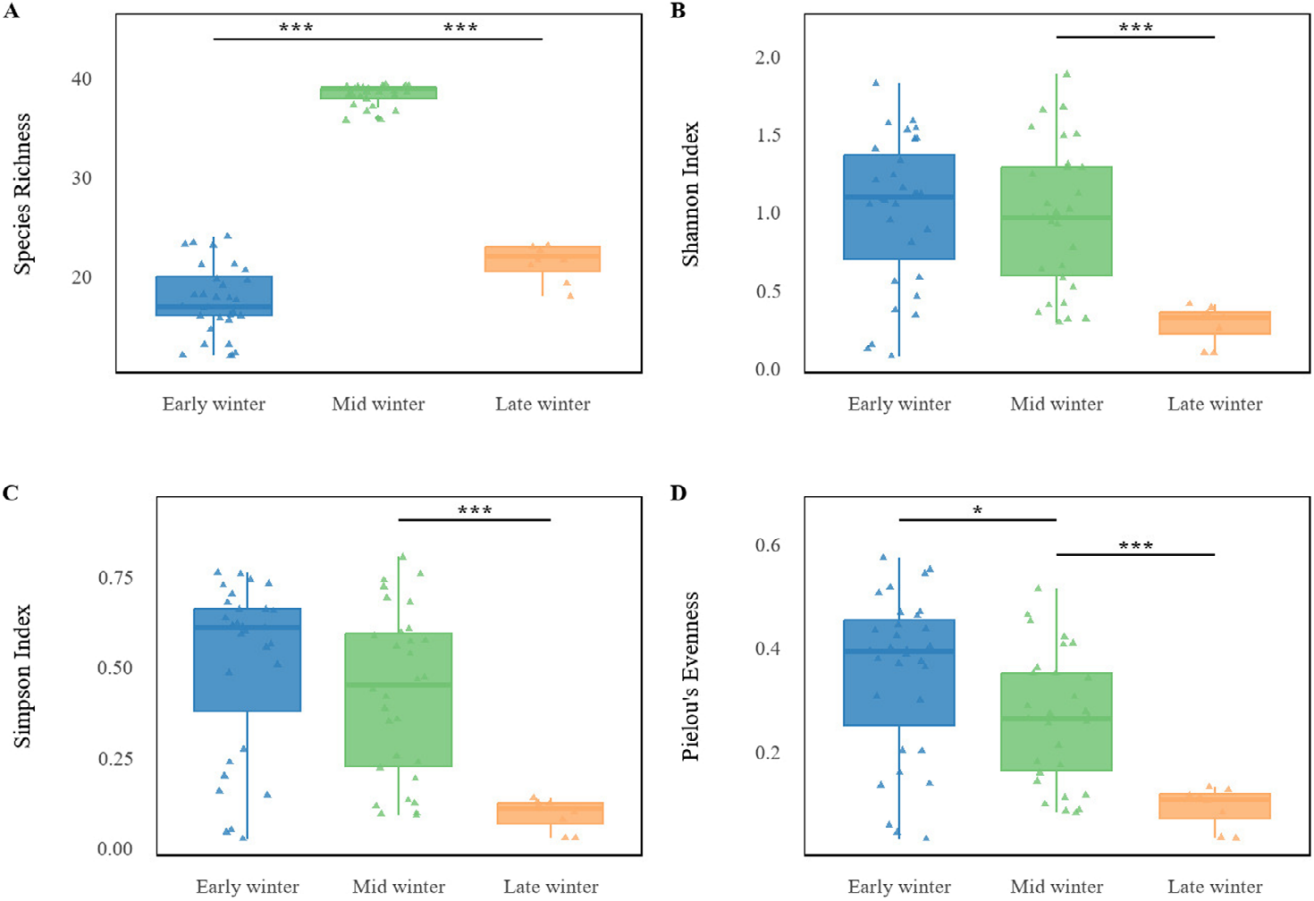
Changes in four α‐diversity indices of the Common Crane diet across the early, mid, and late wintering periods, including species richness (A), Shannon diversity (B), Simpson diversity (C), and Pielou's evenness (D). Asterisks (*) indicate significant differences between stages.

**FIGURE 6:**
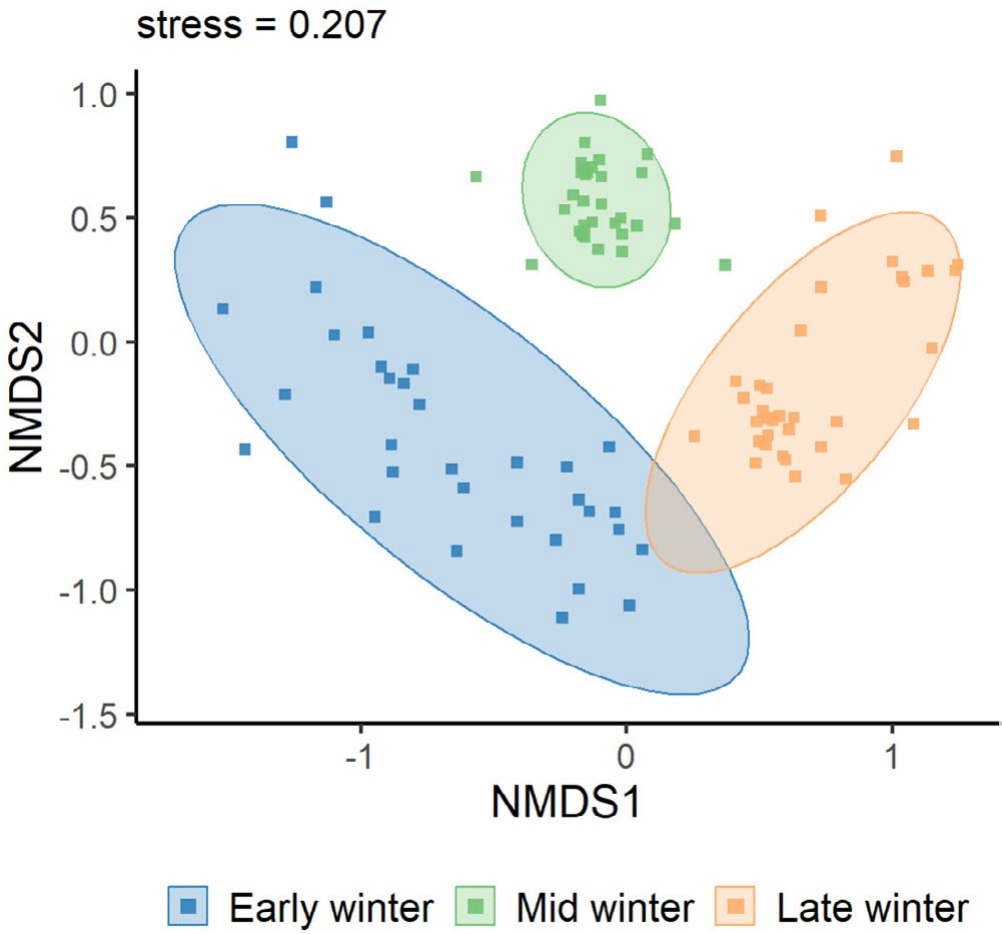
Nonmetric multidimensional scaling (NMDS) based on Bray–Curtis dissimilarity. Different colors represent the wintering stages of Black‐necked Cranes: Early winter (dark blue), mid‐winter (light blue), and late winter (orange).

**FIGURE 7:**
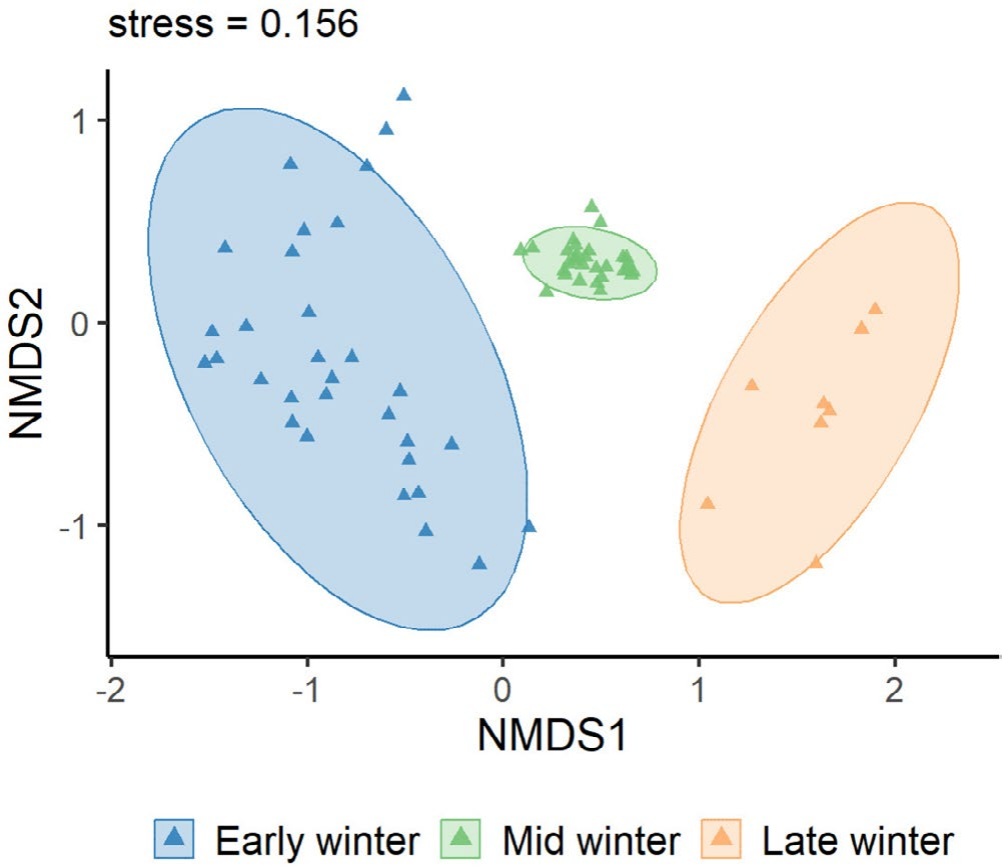
Nonmetric multidimensional scaling (NMDS) ordination based on Bray–Curtis dissimilarity. Different colors represent the three wintering periods of the Common Crane: Early wintering (dark blue), mid‐wintering (light blue), and late wintering (orange).

Black‐necked Cranes displayed a relatively conservative feeding strategy, with diets dominated by Poaceae (40.67% ± 37.26%), Solanaceae (20.70% ± 29.74%), and Cyperaceae (11.66% ± 26.26%), and Polygonaceae (9.71% ± 18.83%) as supplementary intake (Table A1). Core dietary species included *Solanum tuberosum* , *Poa annua* , *Deyeuxia pyramidalis* , *Cyperus serotinus* , and *Fagopyrum esculentum* . Agricultural crops and noncrop plants contributed roughly equal proportions. In contrast, Common Cranes exhibited a more dynamic dietary pattern, dominated by Poaceae (43.67% ± 37.18%), Polygonaceae (31.98% ± 34.95%), and Asteraceae (8.53% ± 20.76%), with *Fagopyrum esculentum* , *Poa annua* , *Miscanthus floridulus* , *Zea mays* , and *Helianthus annuus* as core taxa (Table A2). Agricultural and wild plants contributed comparable proportions. Across winter stages, species richness was consistently higher in Black‐necked Cranes than in Common Cranes (43 vs. 31 in early, 46 vs. 39 in mid, and 39 vs. 27 in late winter; all *p* < 0.001), indicating broader dietary breadth in Black‐necked Cranes (Figure 3). Alpha‐diversity analyses showed that Black‐necked Crane richness peaked in mid‐winter (*p* < 0.001), while Shannon, Simpson, and Pielou's indices remained stable (Figure 4). In Common Cranes, richness increased significantly from early to mid‐winter (*p* < 0.001) but declined in late winter, accompanied by a parallel decline in evenness from mid to late winter (Figure 5). Beta‐diversity analyses for both species revealed clear temporal separation among winter stages (Figures 6 and 7).

Seasonal dietary shifts were evident in both species. In early winter, Black‐necked Crane diets were dominated by Poaceae (37.33% ± 37.14%) and Polygonaceae (21.67% ± 26.79%), with *Poa annua* , *Fagopyrum esculentum* , *Solanum tuberosum* , and *Deyeuxia pyramidalis* as major foods, and agricultural crops accounting for 33.36% ± 36.17%. In mid‐winter, diets became strongly Poaceae‐dominated (61.55% ± 34.47%), with *Deyeuxia pyramidalis* (29.83% ± 37.84%), *Poa annua* (27.20% ± 32.40%), and *Cyperus serotinus* as key species, while *Fagopyrum esculentum* declined markedly; agricultural items dropped to 12.46% ± 15.77%, indicating heavy reliance on natural meadow resources. In late winter, as meadows degraded and agricultural residues reappeared, Black‐necked Cranes shifted toward crop‐derived foods, with Solanaceae (37.42% ± 36.07%), Poaceae (24.38% ± 31.58%), and Cyperaceae (18.30% ± 35.70%) as major contributors, reflecting a transition from meadow plants to agricultural resources. Common Crane diets also varied substantially across winter. In early winter, diets were dominated by Poaceae (63.16% ± 35.38%), with *Poa annua* , *Fagopyrum esculentum* , *Miscanthus floridulus* , and *Zea mays* as major foods, and agricultural items constituting 39.24% ± 28.71%. In mid‐winter, diets shifted sharply toward Polygonaceae (46.50% ± 33.59%), dominated by *Fagopyrum esculentum* (45.54% ± 33.30%), while *Poa annua* remained important and agricultural plants increased to 49.76% ± 31.73%. In late winter, diets transitioned to Asteraceae (50.08% ± 41.37%) and Cyperaceae (25.69% ± 44.68%), dominated by *Helianthus annuus* , *Eleocharis* spp., and *Fagopyrum esculentum* , with agricultural items increasing to 62.38% ± 38.61%, reflecting a seasonal return to farmland resources.

**FIGURE 8:**
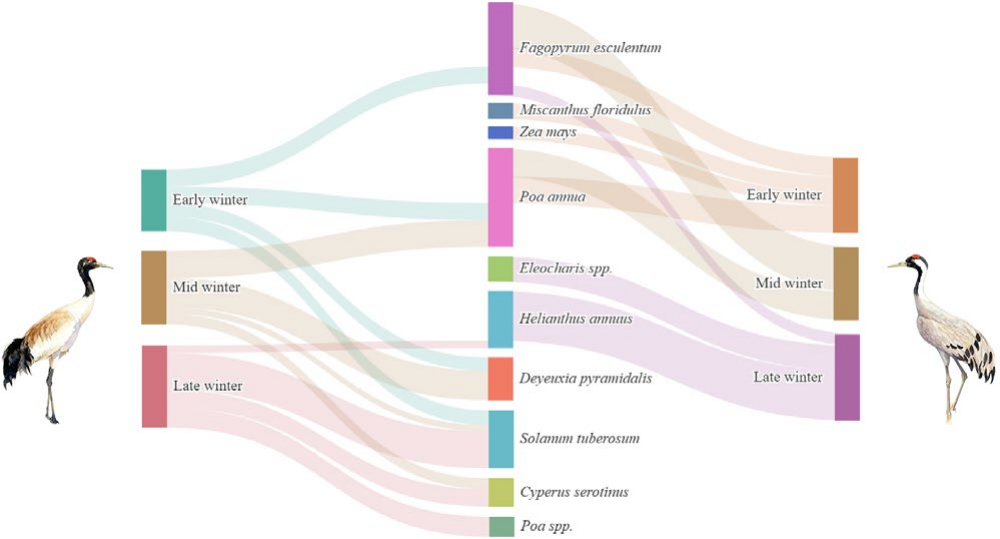
Dietary composition of Black‐necked Cranes (left) and Common Cranes (right) at the species level during early, mid, and late wintering periods. The widths of connecting bands represent the relative abundances of each food item across different periods (all > 5%).

Comparisons between species showed no significant differences in food diversity or evenness in early and mid‐winter (all *p* > 0.05) (Figure 8). By late winter, however, Black‐necked Cranes exhibited significantly higher Shannon (1.82 vs. 1.12), Simpson (0.76 vs. 0.60), and Pielou's evenness (0.50 vs. 0.34) values (all *p* < 0.001), suggesting that Black‐necked Cranes maintained higher dietary diversity under competitive conditions. Overall, Black‐necked Cranes showed a more stable and broad‐spectrum feeding strategy, whereas Common Cranes displayed pronounced stage‐specific dietary shifts, transitioning from Poaceae‐dominated diets in early winter to Polygonaceae in mid‐winter and Asteraceae/Cyperaceae in late winter.

### 3.3 Nutritional Niches Overlap and Differentiation

Throughout the wintering period, Black‐necked Cranes and Common Cranes exhibited fundamentally different foraging strategies. Although both species occasionally occurred in overlapping areas, their foraging habitats differed: Black‐necked Cranes consistently used farmland around 2500 m, whereas Common Cranes foraged across a broader elevational range, including mid‐slope and higher‐altitude sites (Figures 1 and 2). Black‐necked Cranes followed a conservative and gradual adaptive pattern, consistently relying on core taxa from Poaceae, Solanaceae, and Cyperaceae (Table A1). In contrast, Common Cranes adopted a specialized and dynamic pattern, with core food items shifting completely across wintering stages (Table A2). This divergence resulted in clear differentiation in primary plant composition (> 5% proportion) between the two species (Figure 8).

In early winter, both species mainly consumed *Poa annua* and *Fagopyrum esculentum* (Tables A1 and A2). Black‐necked Cranes primarily fed on *Fagopyrum esculentum* , *Poa annua* , *Solanum tuberosum* , and *Deyeuxia pyramidalis* , whereas Common Cranes focused on *Poa annua* and *Fagopyrum esculentum* , supplemented by *Miscanthus floridulus* and *Zea mays* . This dietary similarity occurred despite partial spatial segregation, as Common Cranes mainly used higher‐elevation sites.

By mid‐winter, *Poa annua* remained a shared primary food, but dietary differentiation was evident: Black‐necked Cranes shifted toward *Deyeuxia pyramidalis* and added *Cyperus serotinus* while reducing *Fagopyrum esculentum* intake, whereas Common Cranes relied heavily on *Fagopyrum esculentum* , with other items disappearing from the main diet. During this period, Common Cranes expanded their foraging range downslope, increasing spatial contact with Black‐necked Cranes.

In late winter, the diets of the two species diverged completely. Black‐necked Cranes mainly consumed *Solanum tuberosum* , *Poa* spp., *Cyperus serotinus* , and *Helianthus annuus* , whereas Common Cranes fed primarily on agricultural crops, with *Helianthus annuus* and *Eleocharis* spp. accounting for 75.64% of their diet and *Fagopyrum esculentum* becoming secondary. These results indicate that the two species initially shared similar diets in early winter, but gradually diverged, culminating in completely distinct strategies prior to migration.

Over winter, NO declined steadily from 0.602 in early winter to 0.475 in mid‐winter and further to 0.145 in late winter, indicating that Black‐necked Cranes successfully achieved ecological niche separation from Common Cranes. ND showed the opposite trend, increasing from 0.398 (early) to 0.525 (mid) and reaching 0.855 (late), further revealing the competitive interactions between the two crane species. Throughout the wintering period, Common Cranes consistently exhibited broader niche widths than Black necked Cranes, indicating a higher degree of foraging flexibility (0.177 vs. 0.139 in early, 0.361 vs. 0.219 in mid, and 0.406 vs. 0.236 in late winter). The expansion of niche width in both species as winter progressed suggests increasing resource constraints and intensified interspecific interactions.

**FIGURE 9:**
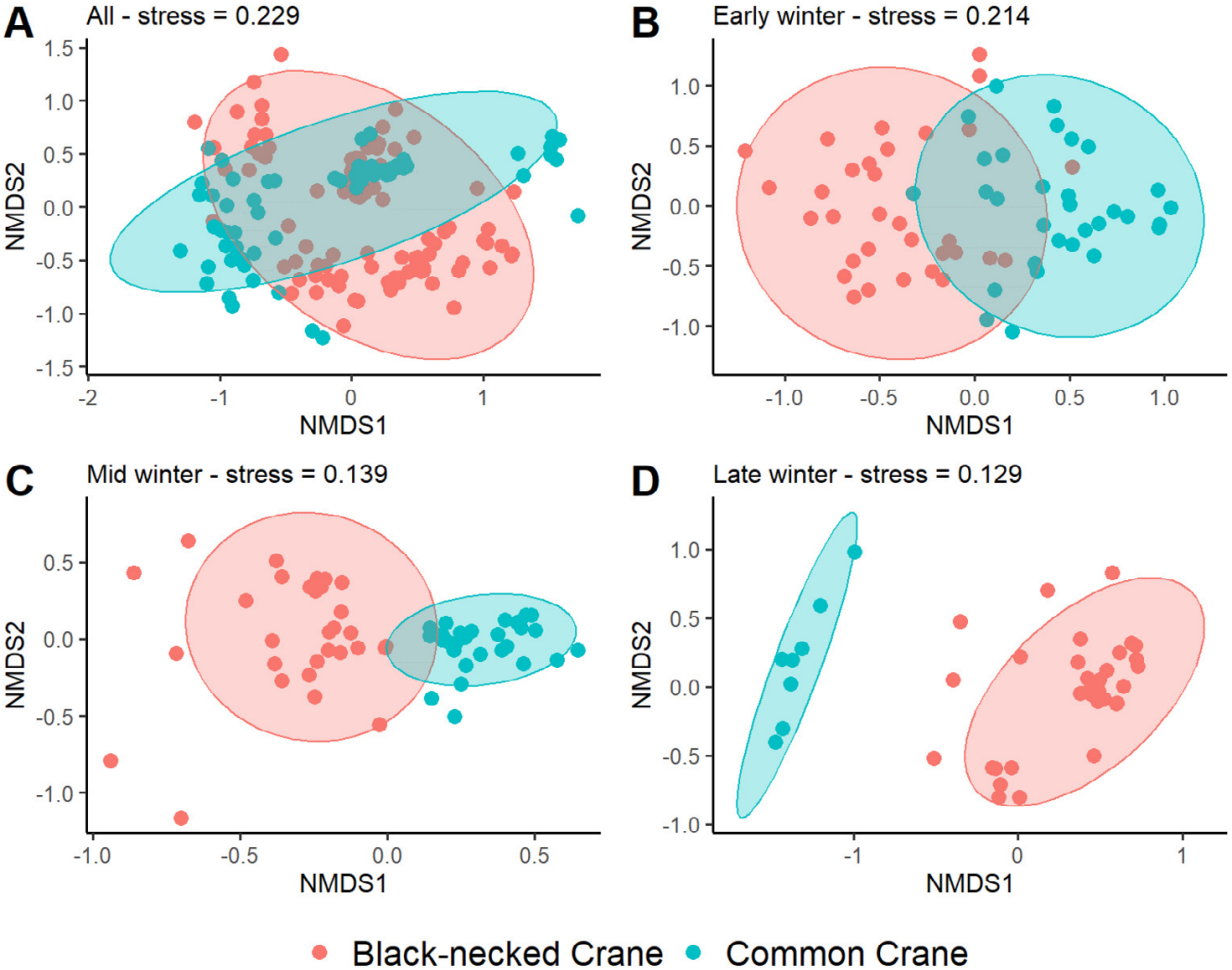
Nonmetric multidimensional scaling (NMDS) based on Bray–Curtis dissimilarity. Red points represent Black‐necked Cranes, and green points represent Common Cranes. Panels show the overall wintering period (A), early winter (B), mid‐winter (C), and late winter (D).

NMDS analysis provided a reliable ordination of dietary composition across the wintering period. Stress values decreased from 0.214 in early winter to 0.129 in late winter (Figure 9), all well below the 0.3 threshold, indicating that the ordination results were robust. Together with the observed spatial patterns, these results show that Black‐necked Cranes, under competitive pressure from Common Cranes, gradually increased dietary specialization, ultimately transitioning from high niche overlap to pronounced niche separation.

## 4 Discussion

### 4.1 Foraging Habitat Use of BNC and CC

Regarding spatial use, Black‐necked Cranes showed high niche conservatism throughout the wintering period. They mainly concentrated their foraging in core wetlands and surrounding farmland at an elevation of approximately 2500 m, with little fluctuation in their range. This stable, low‐elevation strategy is consistent with previous findings that Black‐necked Cranes exhibit strong site fidelity and rely heavily on farmland–wetland mosaics adjacent to roosting sites, allowing them to minimize movement costs and maintain predictable access to food resources (Kong et al. 2018; Zhang et al. 2021). In contrast, Common Cranes foraged at a higher average elevation of around 2700 m and showed greater variability in spatial use, significantly exceeding the activity range of Black‐necked Cranes. Their elevational envelope spanned from low‐lying farmland to higher, more topographically heterogeneous slopes, reflecting a more flexible habitat‐use strategy commonly reported for this species in other wintering regions.

This vertical spatial segregation forms a key mechanism that enables their stable coexistence, as the two species partition the elevational gradient and associated resource distributions. However, during mid‐winter, resource scarcity prompted Common Cranes to expand their foraging range, leading them to partially enter the lower‐elevation core areas of Black‐necked Cranes. This seasonal contraction of spatial segregation aligns with patterns observed in multi‐species crane assemblages, where harsher winter conditions can temporarily intensify interspecific interactions (Son et al. 2025). Such stage‐specific overlap increased energy expenditure and reduced foraging efficiency for Black‐necked Cranes, potentially affecting their pre‐migratory energy reserves (Wu et al. 2020; Zhang et al. 2021; Wang, Zhang, et al. 2024). The observed patterns therefore highlight a dynamic balance between spatial niche differentiation and seasonal resource‐driven convergence, emphasizing the importance of maintaining heterogeneous farmland–wetland landscapes to support the coexistence of both crane species.

### 4.2 Dietary Spretrum and Variation in Diet of BNC and CC at Various Stages of Winter

Using noninvasive fecal sampling with DNA barcoding and high‐throughput sequencing, our study systematically characterized the dietary variation of wintering Black‐necked and Common Cranes. Our results reveal that the food composition of both species exhibits pronounced temporal fluctuations. Climatic conditions, agricultural activities that affect resource availability, and the cranes' own flexible responses to environmental variation all influence these changes (Dong et al. 2016; Hou, Li, et al. 2021; Ma et al. 2024). These findings are consistent with studies from Poyang Lake, where cranes restructure their diets in response to submerged plant decline. Similarly, both species in our study adjust their feeding strategies according to resource availability (Hou, Li, et al. 2021).

The Black‐necked Crane adopted a relatively conservative wintering strategy. It primarily consumed plant‐based foods, showing a consistent reliance on Poaceae, Solanaceae, and Cyperaceae, supplemented by Polygonaceae. Its diet composition changed in distinct phases. In early winter, post‐harvest farmland residues and natural grassland plants provided a diverse food base, allowing the cranes to maintain a broad diet and high foraging flexibility (Li et al. 2024; Dong et al. 2016). Here, their diet was dominated by Poaceae and Polygonaceae. In mid‐winter, as decreasing temperatures and snowfall reduced surface resources, Black‐necked Cranes expanded their foraging range. They began to rely primarily on natural grassland species to maintain energy and nutritional balance, with Poaceae dominating their intake. By late winter, the cranes adopted a more concentrated diet, focusing on a mixture of energy‐rich crops like *Solanum tuberosum* (from Solanaceae) alongside Poaceae and Cyperaceae, reflecting a pre‐migratory energy storage strategy. This transition from a diverse early diet to a concentrated, energy‐rich late‐winter diet illustrates the species' dynamic response to environmental changes and energy demands, highlighting its dietary flexibility and adaptability.

Previous studies confirm that Black‐necked Crane diets vary geographically, largely reflecting landscape composition and resource availability. For instance, in the Caohai wetlands of Guizhou, which feature abundant aquatic plants, Cyperaceae dominates their diet (76.5%) (Li et al. 1997). In contrast, the highland farmland–wetland mosaics of Dashanbao, Yunnan, and our study area in Huize support more diverse diets that include natural herbaceous plants (Poaceae, Solanaceae, Polygonaceae) and crops such as cereals and *Solanum tuberosum* (Liu et al. 2014; Dong et al. 2016; Li et al. 2024). In Bhutan's Bumdeling wintering grounds, Black‐necked Cranes rely heavily on post‐harvest cereals and herbaceous plants, reflecting landscape‐driven dietary concentration in agricultural valleys (Wangchuk et al. 2025). These patterns indicate that landscape type strongly shapes the Black‐necked Crane's diet structure. Lake wetlands favor a concentrated consumption of Cyperaceae, farmland–wetland mosaics promote dietary diversity, and crop‐dominated landscapes drive a reliance on high‐energy residues. Our study shows that in Huize, Black‐necked Cranes adapt to dynamic resources through early broad‐spectrum feeding, mid‐winter diversification, and late‐winter energy‐focused foraging, which closely aligns their diet structure with landscape resource patterns.

In contrast, the Common Crane showed stronger dietary specialization and spatiotemporal adaptability. Its diet also changed in phases. In early winter, it consumed a diet dominated by Poaceae and residual crops. In mid‐winter, it relied heavily on *Fagopyrum esculentum* . In late winter, it shifted to *Helianthus annuus* and *Eleocharis* spp. This progression reveals a gradual taxonomic transition from Poaceae to Polygonaceae, Asteraceae, and Cyperaceae. This sequential focus on a limited set of high‐energy plants reflects the Common Crane's sensitivity to seasonal food distribution and its spatiotemporal adaptation. Other cranes and large waterbirds exhibit similar stage‐specific foraging strategies. For example, White Cranes at Poyang Lake shift from submerged plants to post‐harvest rice and tubers when water levels drop (Hou, Li, et al. 2021).

Studies across different landscapes further illustrate the Common Crane's dietary flexibility. In the coastal wetland–farmland mosaic of Yancheng National Nature Reserve, Jiangsu, cranes demonstrate high plasticity by switching between provisioned food (e.g., *Zea mays* , *Triticum aestivum* ) and natural plants (Poaceae, Cyperaceae) (Zhao et al. 2023). In the reservoir–farmland–forest landscape of Beijing's Wild Duck Lake and Miyun Reservoir, their diet consists mainly of cereals such as *Zea mays* , *Sorghum bicolor* , *Glycine max* , and *Triticum aestivum* , reflecting a dependence on farmland residues (Zhan et al. 2007). In southern Russia, Common Cranes spend considerable time feeding on post‐harvest cereals, indicating a strong adaptation to agricultural resources (Kondrakova 2023). In Huize, Yunnan, they preferentially forage along farmland and wetland margins, consuming both crop residues and herbaceous plants (Kong et al. 2018). Collectively, these findings suggest that Common Cranes employ a strategy of stage‐specific concentrated feeding in response to spatiotemporal resource fluctuations, with landscape composition largely driving dietary differences.

Physiological regulation also contributes to these dietary shifts. For example, Zhang et al. (2025) showed that the gut microbiota of Black‐necked Cranes dynamically adjusts in concert with their diet, which enhances their adaptation to highland environments and resource fluctuations. Across the wintering period, Black‐necked Cranes consistently exhibited higher food richness (SR) and maintained greater dietary evenness in late winter than Common Cranes, reflecting their generalist advantage under fluctuating resource conditions. Common Cranes, however, sequentially focus on a few high‐energy plants, a strategy that ensures stable energy intake and high foraging efficiency. Together, these findings reveal the adaptive mechanisms cranes use to respond to seasonal environmental variation. Black‐necked Cranes maintain energy balance through early‐winter dietary diversity and late‐winter concentrated feeding, whereas Common Cranes adopt a strategy of stage‐specific concentrated feeding. These patterns provide insight into the resource‐use strategies of large waterbirds in agro‐wetland ecosystems.

### 4.3 Nutritional Niches Overlap and Differentiation

Regarding spatial use, Black‐necked Cranes showed high niche conservatism throughout the wintering period. They mainly concentrated their foraging in core wetlands and surrounding farmland at an elevation of 2500 m, with little fluctuation in their range. In contrast, Common Cranes foraged at a higher average elevation of 2700 m and showed greater variability in spatial use, significantly exceeding the activity range of Black‐necked Cranes. This vertical spatial segregation forms a key mechanism that enables their stable coexistence. However, during mid‐winter, resource scarcity prompted Common Cranes to expand their foraging range, leading them to partially enter the lower‐elevation core areas of Black‐necked Cranes. This stage‐specific overlap increased energy expenditure and reduced foraging efficiency for Black‐necked Cranes, potentially affecting their pre‐migratory energy reserves.

Diet composition also reflects niche differentiation. In early winter, both species primarily consumed *Poa annua* and *Fagopyrum esculentum* , resulting in high dietary overlap. As winter progressed, Common Cranes consumed significantly more *Poa annua* than Black‐necked Cranes, intensifying competition. Black‐necked Cranes responded by increasing their consumption of alternative resources such as *Deyeuxia pyramidalis* and *Cyperus serotinus* in mid‐winter, which alleviated competitive pressure. Meanwhile, Common Cranes adopted a highly concentrated feeding strategy, relying heavily on *Fagopyrum esculentum* and later shifting to other high‐energy foods like *Helianthus annuus* and *Eleocharis* spp. To meet their pre‐migratory energy demands, Black‐necked Cranes increased their intake of *Solanum tuberosum* in late winter. Overall, Common Cranes maintained a competitive advantage through a “concentrated high‐energy feeding” strategy, whereas Black‐necked Cranes employed a “diverse diet” strategy, reflecting a more passive, flexible response.

Both species also exhibited adaptive use of specific plant resources to meet energy requirements. They consumed seeds of Polygonaceae, which are high in tannins, and dug up rhizomes of Cyperaceae, which contain alkaloids (Li et al. 1997; Zhao et al. 2023). Although these secondary metabolites typically deter most waterbirds, cranes can tolerate and efficiently digest them. A co‐evolved gut microbiota, capable of breaking down cellulose and metabolizing these anti‐nutritional compounds, likely facilitates this ability (Kohl et al. 2014; Li et al. 2023). This specialized digestion enhances their energy acquisition and nutritional balance and may improve winter survival.

Niche metrics further confirmed this dynamic differentiation. NO declined from 0.602 in early winter to 0.475 in mid‐winter and further to 0.145 in late winter, indicating that Black‐necked Cranes successfully achieved ecological niche separation. By late winter, the nutritional niches of Black‐necked and Common Cranes had fully separated. ND showed the opposite trend, increasing from 0.398 (early) to 0.525 (mid) and reaching 0.855 (late), reflecting a generalist's advantage when resources fluctuate. Although Common Cranes consistently exhibited broader niche breadth than Black‐necked Cranes throughout the wintering period, the pronounced expansion in mid‐winter indicates intensified resource exploitation, likely associated with increased interspecific competition following their downslope range expansion. Both species broadened their niche breadth as winter progressed, but through different mechanisms: Black‐necked Cranes expanded resource use by incorporating additional complementary food items, whereas Common Cranes did so by sequentially concentrating on different dominant resources across stages. These patterns indicate that both species responded to increasing resource limitations by diversifying resource use, albeit to different extents.

In summary, Black‐necked Cranes dominated core wetland resources through strong site fidelity and stable foraging strategies, while Common Cranes compensated by expanding their foraging space and concentrating on energy‐rich foods. This interaction results in a coexistence pattern of “shared primary resources but differentiated use of other resources” (Wu et al. 2020; Zhang et al. 2021; Yanco et al. 2024). Researchers have observed similar mechanisms of dietary differentiation in multiple taxa. For instance, in Poyang Lake wetlands, Hooded Crane ( *G. monacha* ), Common Crane, White Crane ( *G. vipio* ), and Siberian Cranes (*Leucogeranus leucogeranus*) maintained coexistence after the collapse of submerged plant resources by partitioning their diets and adjusting their ecological niches (Hou, Li, et al. 2021). Hooded Cranes relied on stable farmland resources with conservative foraging. Common Cranes expanded their foraging to utilize residual crops and wetland edges. White Cranes shifted to aquatic plants and benthic organisms when resources were scarce. Siberian Cranes preferred shallow, vegetated wetland zones to reduce dietary overlap. These findings highlight that niche differentiation is a crucial mechanism for closely related species to achieve ecological separation and stable coexistence. Morphology, foraging behavior, and resource availability over time shape this process (Gobin et al. 2022; Dai et al. 2023; Guo et al. 2024).

Our study demonstrates that dietary and spatial differentiation between Black‐necked and Common Cranes reflects their dynamic adaptive responses to competitive pressures. Black‐necked Cranes maintain flexibility through a diversified diet, whereas Common Cranes rely on energy‐concentrated feeding to sustain a competitive advantage. As resources declined and population density increased through the winter, their niches shifted from high overlap in the early season to clear separation in the mid‐ and late seasons. Conversely, in resource‐abundant or low‐density systems, niche overlap may increase and competition may weaken, indicating that coexistence is a dynamic response to environmental pressures. Future research should explore how climate change and human activities reshape resource landscapes and species interactions, which will ultimately affect ecosystem stability (Hardy et al. 2017). We must note a potential limitation of this study. We collected only eight Common Crane samples in late winter because most individuals had already migrated. This small sample size may affect our assessment of their late‐winter dietary specialization. Future sampling efforts should better align with the species' phenology and migration patterns to address this.

## 5 Conclusion

Black‐necked Cranes and Common Cranes display distinct nutritional niches and resource‐use strategies during winter. Black‐necked Cranes maintain high niche conservatism, whereas Common Cranes substantially expand their activity range during mid‐winter when resources are scarce, frequently entering the core areas of Black‐necked Cranes. This intrusion reduces foraging efficiency and energy accumulation for Black‐necked Cranes, resulting in a coexistence strategy characterized as “shared basic resources—partitioned specialized resources.” This pattern reflects niche compression and displacement of Black‐necked Cranes under competitive pressure from Common Cranes. Dynamic studies of diet and nutritional niches are therefore critical for informing targeted conservation measures and assessing habitat suitability. Effective management should focus on maintaining the integrity, complexity, and dynamic balance of the wintering ecosystem. First, the stable availability of shared core resources—such as *Solanum tuberosum* , *Poa annua* , and *Fagopyrum esculentum* —must be ensured, particularly during early winter when key food resources are critical. Second, habitats supporting niche differentiation should be protected, including areas containing specialized resources such as *Deyeuxia pyramidalis* and *Cyperus serotinus* , while maintaining foraging sites across different elevation gradients to accommodate spatial segregation. Finally, ecosystem processes should be preserved, with conservation interventions aligned with temporal resource fluctuations. In mid‐winter, when snow cover reduces food availability and competition intensifies, supplemental feeding may be necessary, prioritizing the energy requirements of Black‐necked Cranes at each stage. Integrating these findings into habitat management provides a robust scientific foundation for ensuring the stable survival of Black‐necked Cranes in agricultural landscapes.

## Author Contributions

**Jiayi Wu:** data curation (lead), formal analysis (lead), investigation (equal), methodology (equal), software (lead), visualization (lead), writing – original draft (lead), writing – review and editing (equal). **Yuhui Si:** conceptualization (equal), methodology (equal), supervision (equal), validation (equal). **Xiaoxuan Shang:** investigation (equal), validation (equal). **Chao Li:** investigation (equal), validation (equal), visualization (equal). **Qiang Liu:** conceptualization (lead), funding acquisition (lead), investigation (equal), methodology (equal), project administration (lead), resources (lead), supervision (lead), validation (lead), writing – review and editing (equal).

## Funding

The Yunnan Province Science and Technology Plan Project (202301BD070001‐232) funded the study.

## Conflicts of Interest

The authors declare no conflicts of interest.

## Data Availability

The DNA metabarcoding data generated for this study are available on DRYAD (https://doi.org/10.5061/dryad.jsxksn0q5).

## Appendix A

**TABLE A1:** Diet composition and proportions of Black‐necked Cranes during early, mid, and late wintering stages.

Family	Genus	Species	RRA (%)		
			November (*n* = 32)	January (*n* = 31)	March (*n* = 34)
Melanthiaceae	*Ypsilandra*	*Ypsilandra yunnanensis*	0.02 ± 0.08	0	0
Amaranthaceae	*Acroglochin*	*Acroglochin persicarioides*	0.42 ± 1.40	0.15 ± 0.73	0.03 ± 0.07
Amblystegiaceae	*Drepanocladus*	*Drepanocladus aduncus*	0.65 ± 2.93	0	0.11 ± 0.40
Apiaceae	*Conioselinum*	*Conioselinum pteridophyllum*	1.27 ± 2.22	0.74 ± 3.08	0.28 ± 0.36
Araliaceae	*Hydrocotyle*	*Hydrocotyle sibthorpioides*	0	0.05 ± 0.19	0
Asteraceae	*Pseudognaphalium*	*Pseudognaphalium hypoleucum*	1.67 ± 6.57	2.11 ± 4.81	0.07 ± 0.29
Asteraceae	*Galinsoga*	*Galinsoga parviflora*	2.09 ± 2.64	0.65 ± 1.47	0.06 ± 0.26
Asteraceae	*Senecio*	*Senecio vulgaris*	0	0.05 ± 0.15	0
Asteraceae	*Crassocephalum*	*Crassocephalum crepidioides*	0.06 ± 0.21	0	0
Asteraceae	*Helianthus*	*Helianthus annuus*	0	0	7.80 ± 17.26
Asteraceae	*Gymnanthemum*	*Gymnanthemum amygdalinum*	0	0	0.03 ± 0.14
Brassicaceae	*Cardamine*	*Cardamine flexuosa*	0.05 ± 0.20	0.66 ± 3.40	0
Brassicaceae	*Raphanus*	*Raphanus sativus*	0	0.11 ± 0.61	0
Brassicaceae	*Brassica*	*Brassica rapa*	0.03 ± 0.13	0.02 ± 0.10	0.10 ± 0.26
Brassicaceae	*Brassica*	Unknow	0	0.02 ± 0.04	0
Brassicaceae	*Brassica*	*Brassica oleracea*	0	0	0.21 ± 0.53
Bryaceae	*Bryum*	*Bryum argenteum*	0.02 ± 0.07	0.09 ± 0.19	0.15 ± 0.71
Caryophyllaceae	*Stellaria*	*Stellaria media*	0.53 ± 1.40	0.53 ± 2.80	2.77 ± 15.45
Caryophyllaceae	*Spergula*	*Spergula arvensis*	0.02 ± 0.05	0	0
Coriariaceae	*Coriaria*	*Coriaria napalensis*	0	0	0.02 ± 0.05
Cupressaceae	*Cunninghamia*	*Cunninghamia lanceolata*	0	0.03 ± 0.15	0
Cyperaceae	*Cyperus*	*Cyperus serotinus*	4.59 ± 14.10	10.96 ± 22.14	17.72 ± 35.42
Cyperaceae	*Eleocharis*	Unknow	0	0.69 ± 2.82	0.50 ± 0.30
Cyperaceae	*Cyperus*	*Cyperus cyperoides*	0.02 ± 0.04	0	0.09 ± 0.22
Ditrichaceae	*Ditrichum*	*Ditrichum heteromallum*	0	0.29 ± 1.12	0.23 ± 0.98
Fabaceae	*Parochetus*	*Parochetus communis*	1.01 ± 5.60	0.14 ± 0.66	0.05 ± 0.04
Fabaceae	*Trifolium*	*Trifolium repens*	0.73 ± 1.87	0.03 ± 0.12	0.05 ± 0.04
Fabaceae	*Vicia*	*Vicia bungei*	0.06 ± 0.29	0	0.04 ± 0.03
Geraniaceae	*Geranium*	*Geranium sibiricum*	0.06 ± 0.30	0	0
Juncaceae	*Juncus*	*Juncus effusus*	0.40 ± 1.75	0.02 ± 0.07	0.05 ± 0.04
Lamiaceae	*Mentha*	*Mentha × piperita*	0.10 ± 0.15	0.17 ± 0.68	0.10 ± 0.33
Lamiaceae	*Elsholtzia*	Unknow	0	0.04 ± 0.05	0
Lamiaceae	*Lavandula*	Unknow	0.06 ± 0.08	0.01 ± 0.01	0
Lamiaceae	*Elsholtzia*	*Elsholtzia ciliata*	0.04 ± 0.07	0	0
Mazaceae	*Mazus*	*Mazus pumilus*	0	0.04 ± 0.13	0
Orobanchaceae	*Pedicularis*	*Pedicularis oederi*	0.03 ± 0.15	0.06 ± 0.19	0
Plantaginaceae	*Veronica*	*Veronica serpyllifolia*	0.02 ± 0.08	0.30 ± 1.62	0
Plantaginaceae	*Hippuris*	*Hippuris vulgaris*	0	0.08 ± 0.04	0
Plantaginaceae	*Veronica*	*Veronica polita*	0	0	0.04 ± 0.23
Plantaginaceae	*Callitriche*	*Callitriche palustris*	0	0	0.03 ± 0.17
Poaceae	*Deyeuxia*	*Deyeuxia pyramidalis*	13.56 ± 28.41	29.83 ± 37.84	0.47 ± 0.83
Poaceae	*Poa*	*Poa annua*	17.10 ± 32.76	27.20 ± 32.40	0.38 ± 1.06
Poaceae	*Miscanthus*	*Miscanthus floridulus*	2.58 ± 4.84	2.18 ± 3.74	1.68 ± 3.91
Poaceae	*Zea*	*Zea mays*	1.36 ± 3.52	1.89 ± 4.25	1.51 ± 3.62
Poaceae	*Agrostis*	*Agrostis stolonifera*	0.25 ± 0.49	0.13 ± 0.16	0.02 ± 0.06
Poaceae	*Arrhenatherum*	*Arrhenatherum elatius*	0	0.11 ± 0.21	0
Poaceae	*Elymus*	*Elymus durus*	0	0.06 ± 0.23	0
Poaceae	*Polypogon*	*Polypogon fugax*	0	0.06 ± 0.09	0
Poaceae	*Eriochloa*	*Eriochloa villosa*	0	0.02 ± 0.09	0
Poaceae	*Alopecurus*	*Alopecurus japonicus*	0.02 ± 0.04	0.02 ± 0.03	0.03 ± 0.05
Poaceae	*Triticum*	*Triticum aestivum*	0	0.01 ± 0.06	0
Poaceae	*Elymus*	*Elymus dahuricus*	2.35 ± 7.07	0	0
Poaceae	*Phaenosperma*	*Phaenosperma globosum*	0.04 ± 0.17	0	0
Poaceae	*Poa*	Unknow	0.02 ± 0.09	0	20.27 ± 30.99
Polygonaceae	*Fagopyrum*	*Fagopyrum esculentum*	17.11 ± 27.36	3.99 ± 6.60	3.49 ± 14.32
Polygonaceae	*Persicaria*	*Persicaria nepalensis*	4.10 ± 8.88	0.73 ± 1.81	0.75 ± 1.25
Polygonaceae	*Persicaria*	*Persicaria lapathifolia*	0.26 ± 1.29	0.10 ± 0.26	0.02 ± 0.04
Polygonaceae	*Fagopyrum*	*Fagopyrum gilesii*	0	0.08 ± 0.15	0
Polygonaceae	*Persicaria*	*Persicaria perfoliata*	0	0.02 ± 0.05	0.01 ± 0.02
Polygonaceae	*Persicaria*	*Persicaria viscosa*	0.15 ± 0.64	0	0
Polygonaceae	*Persicaria*	*Persicaria senticosa*	0.02 ± 0.05	0	0
Polygonaceae	*Persicaria*	*Persicaria runcinata*	0.02 ± 0.04	0	0
Polygonaceae	*Rumex*	*Rumex crispus*	0	0	0.02 ± 0.05
Potamogetonaceae	*Potamogeton*	*Potamogeton perfoliatus*	0	0.03 ± 0.01	0.02 ± 0.03
Pottiaceae	*Didymodon*	*Didymodon nigrescens*	0	0.11 ± 0.46	0
Pottiaceae	*Barbula*	*Barbula unguiculata*	0	0	0.01 ± 0.06
Primulaceae	*Lysimachia*	*Lysimachia congestiflora*	0.06 ± 0.25	0	0
Rubiaceae	*Galium*	*Galium aparine*	0	0.04 ± 0.19	0
Rubiaceae	*Galium*	*Galium bungei*	0.13 ± 0.73	0	0
Solanaceae	*Solanum*	*Solanum tuberosum*	14.85 ± 24.59	6.45 ± 13.05	37.32 ± 36.13
Solanaceae	*Lycium*	*Lycium hybrid cultivar*	0.01 ± 0.03	0	0.11 ± 0.26
Melanthiaceae	*Ypsilandra*	*Ypsilandra yunnanensis*	0.02 ± 0.08	0	0
Amaranthaceae	*Acroglochin*	*Acroglochin persicarioides*	0.42 ± 1.40	0.15 ± 0.73	0.03 ± 0.07

**TABLE A2:** Diet composition and proportions of Common Cranes during early, mid, and late wintering stages.

Family	Genus	Species	RRA (%)		
			November (*n* = 30)	January (*n* = 30)	March (*n* = 8)
Melanthiaceae	*Ypsilandra*	*Ypsilandra yunnanensis*	0.02 ± 0.04	0	0
Amaranthaceae	*Acroglochin*	*Acroglochin persicarioides*	0	0.01 ± 0.01	0
Amblystegiaceae	*Drepanocladus*	*Drepanocladus aduncus*	0.10 ± 0.30	0	0.16 ± 0.25
Apiaceae	*Conioselinum*	*Conioselinum pteridophyllum*	0.04 ± 0.14	0.06 ± 0.06	0.03 ± 0.04
Asteraceae	*Galinsoga*	*Galinsoga parviflora*	0.45 ± 0.76	0.30 ± 0.28	0
Asteraceae	*Senecio*	*Senecio vulgaris*	0	0.01 ± 0.02	0
Asteraceae	*Pseudognaphalium*	*Pseudognaphalium hypoleucum*	2.72 ± 3.99	2.51 ± 4.59	0
Asteraceae	*Helianthus*	*Helianthus annuus*	0	0	50.08 ± 41.37
Brassicaceae	*Cardamine*	*Cardamine flexuosa*	0	0.04 ± 0.01	0
Brassicaceae	*Brassica*	Unknow	0.04 ± 0.09	0.02 ± 0.06	0
Bryaceae	*Bryum*	*Bryum argenteum*	0.01 ± 0.04	0.07 ± 0.19	0.04 ± 0.10
Caryophyllaceae	*Stellaria*	*Stellaria media*	0	0.03 ± 0.03	0.05 ± 0.06
Coriariaceae	*Coriaria*	*Coriaria napalensis*	0	0	0.04 ± 0.08
Cyperaceae	*Eleocharis*	Unknow	0	0.10 ± 0.06	25.56 ± 44.58
Cyperaceae	*Cyperus*	*Cyperus serotinus*	0.01 ± 0.07	0.12 ± 0.02	0.12 ± 0.12
Cyperaceae	*Carex*	Unknow	0	0	0.01 ± 0.02
Ditrichaceae	*Ditrichum*	*Ditrichum heteromallum*	0	0.93 ± 2.46	0.27 ± 0.51
Eucommiaceae	*Eucommia*	*Eucommia ulmoides*	0.01 ± 0.06	0	0
Fabaceae	*Trifolium*	*Trifolium repens*	0	0	0.04 ± 0.10
Fabaceae	*Vicia*	*Vicia bungei*	0.21 ± 1.15	0	0
Fabaceae	*Toxicopueraria*	*Toxicopueraria peduncularis*	0.02 ± 0.07	0	0
Fabaceae	*Castanopsis*	*Castanopsis carlesii*	0	0	0.02 ± 0.03
Fabaceae	*Parochetus*	*Parochetus communis*	0	0.01 ± 0.01	0
Hypericaceae	*Hypericum*	*Hypericum ascyron*	0	0.02 ± 0.04	0
Lamiaceae	*Ocimum*	*Ocimum americanum*	0	0.01 ± 0.03	0
Lamiaceae	*Mentha*	*Mentha × piperita*	0	0.04 ± 0.10	0
Lamiaceae	*Elsholtzia*	*Elsholtzia ciliata*	0.10 ± 0.41	0	0.19 ± 0.41
Lamiaceae	*Elsholtzia*	Unknow	0	0.95 ± 3.29	0
Lamiaceae	*Lavandula*	Unknow	0.07 ± 0.16	0.03 ± 0.04	0
Lamiaceae	*Pseudocaryopteris*	*Pseudocaryopteris paniculata*	0	0.04 ± 0.12	0
Orobanchaceae	*Pedicularis*	*Pedicularis cephalantha*	0	0.01 ± 0.05	0
Plantaginaceae	*Scoparia*	*Scoparia dulcis*	0	0.05 ± 0.14	0
Plantaginaceae	*Veronica*	*Veronica anagallis‐aquatica*	0	0	0.02 ± 0.02
Plantaginaceae	*Scoparia*	*Scoparia dulcis*	0	0.12 ± 0.10	0
Plantaginaceae	*Veronica*	*Veronica anagallis‐aquatica*	0	0.01 ± 0.00	0
Poaceae	*Deyeuxia*	*Deyeuxia pyramidalis*	3.22 ± 11.88	1.80 ± 1.89	0
Poaceae	*Polypogon*	*Polypogon fugax*	0.01 ± 0.04	0	0
Poaceae	*Elymus*	*Elymus durus*	0.03 ± 0.10	0.07 ± 0.16	0
Poaceae	*Elymus*	*Elymus dahuricus*	3.38 ± 5.73	0	0
Poaceae	*Elymus*	*Elymus caninus*	0.01 ± 0.03	0	0
Poaceae	*Alopecurus*	*Alopecurus japonicus*	0.01 ± 0.02	0.02 ± 0.05	0
Poaceae	*Miscanthus*	*Miscanthus floridulus*	16.41 ± 19.12	3.11 ± 9.45	0.23 ± 0.24
Poaceae	*Agrostis*	*Agrostis stolonifera*	0.09 ± 0.29	0.02 ± 0.03	0
Poaceae	*Zea*	*Zea mays*	12.82 ± 15.66	1.79 ± 4.90	0.07 ± 0.16
Poaceae	*Cynodon*	*Cynodon dactylon × Cynodon transvaalensis*	0	0	0.16 ± 0.44
Poaceae	*Poa*	*Poa annua*	27.17 ± 35.69	28.71 ± 28.21	0.01 ± 0.01
Poaceae	*Poa*	Unknow	0	0.01 ± 0.02	0.60 ± 0.36
Polygonaceae	*Persicaria*	*Persicaria senticosa*	0.02 ± 0.05	0	0
Polygonaceae	*Persicaria*	*Persicaria sagittata*	0.03 ± 0.15	0	0
Polygonaceae	*Fagopyrum*	*Fagopyrum esculentum*	19.65 ± 28.52	45.55 ± 33.30	11.72 ± 32.36
Polygonaceae	*Persicaria*	*Persicaria perfoliata*	0	0.05 ± 0.12	0
Polygonaceae	*Persicaria*	*Persicaria nepalensis*	3.14 ± 10.29	0.21 ± 0.36	0.04 ± 0.03
Polygonaceae	*Persicaria*	*Persicaria lapathifolia*	0.01 ± 0.05	0	0
Polygonaceae	*Fagopyrum*	*Fagopyrum gilesii*	0	0.62 ± 0.39	0
Polygonaceae	*Rumex*	*Rumex crispus*	0	0.06 ± 0.07	0.01 ± 0.04
Potamogetonaceae	*Potamogeton*	*Potamogeton perfoliatus*	0	0.03 ± 0.01	0.06 ± 0.05
Pottiaceae	*Didymodon*	*Didymodon nigrescens*	0	0.06 ± 0.24	0
Primulaceae	*Myrsine*	*Myrsine semiserrata*	0	0	0.02 ± 0.06
Ranunculaceae	*Ranunculus*	*Ranunculus bungei*	0	0	0.02 ± 0.01
Solanaceae	*Capsicum*	*Capsicum annuum*	0.01 ± 0.03	0	0
Solanaceae	*Solanum*	*Solanum tuberosum*	6.2 ± 12.77	2.41 ± 2.86	0.51 ± 0.28
Solanaceae	*Lycium*	*Lycium hybrid cultivar*	0.01 ± 0.02	0	0
Typhaceae	*Typha*	*Typha latifolia*	0	0	0.07 ± 0.10
